# Rheumatism and Dyspepsia

**Published:** 1875-07

**Authors:** 


RHEUMATISM AND DYSPEPSIA.—Cakes made of arrow-root, spread with butter, and eaten to the exclusion of all other food are reported to have been found effective in the cure of chronic rheumatism and chronic dyspepsia, without medication of any sort. The bowels became regular, pain gradually lessoned and finally disappeared entirely, and the distress indused by eating, no longer existed. Tapioca, cooked without milk or eggs, produced similar results.

The imagination is of so delicate a texture that even words wound it.

The mind wears the colors of the soul as the valet does those of his master.

